# Ocean acidification drives global reshuffling of ecological communities

**DOI:** 10.1111/gcb.16410

**Published:** 2022-09-29

**Authors:** Ivan Nagelkerken, Sean D. Connell

**Affiliations:** ^1^ Southern Seas Ecology Laboratories, School of Biological Sciences and The Environment Institute The University of Adelaide Adelaide South Australia Australia

**Keywords:** biodiversity, climate change, community structure, coral reefs, habitat shifts, mesocosms, rocky reefs, seagrasses, species replacements, volcanic CO_2_ vents

## Abstract

The paradigm that climate change will alter global marine biodiversity is one of the most widely accepted. Yet, its predictions remain difficult to test because laboratory systems are inadequate at incorporating ecological complexity, and common biodiversity metrics have varying sensitivity to detect change. Here, we test for the prevalence of global responses in biodiversity and community‐level change to future climate (acidification and warming) from studies at volcanic CO_2_ vents across four major global coastal ecosystems and studies in laboratory mesocosms. We detected globally replicable patterns of species replacements and community reshuffling under ocean acidification in major natural ecosystems, yet species diversity and other common biodiversity metrics were often insensitive to detect such community change, even under significant habitat loss. Where there was a lack of consistent patterns of biodiversity change, these were a function of similar numbers of studies observing negative versus positive species responses to climate stress. Laboratory studies showed weaker sensitivity to detect species replacements and community reshuffling in general. We conclude that common biodiversity metrics can be insensitive in revealing the anticipated effects of climate stress on biodiversity—even under significant biogenic habitat loss—and can mask widespread reshuffling of ecological communities in a future ocean. Although the influence of ocean acidification on community restructuring can be less evident than species loss, such changes can drive the dynamics of ecosystem stability or their functional change. Importantly, species identity matters, representing a substantial influence of future oceans.

## Graphical abstract

Here, we test for global patterns in biodiversity and community structure change to future climate (ocean acidification and warming), using studies from volcanic CO_2_ vents across four major global coastal ecosystems and studies in laboratory mesocosms. We detected globally replicable patterns of species replacement and community reshuffling under ocean acidification, yet species diversity and other common biodiversity metrics were often insensitive to detect such community change, even under significant habitat loss. Although the influence of ocean acidification on community restructuring can be less evident than net species loss, such reshuffling can alter ecosystem stability and drive functional change.

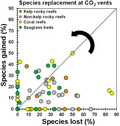

## 1 INTRODUCTION

Research into the ecological consequences of climate change is particularly vexing because no single experiment can simulate future climate, let alone the complexity of the real world today. No ecological study, whether in the laboratory or field, can fully replicate the complex ecological interactions that exist in nature across the time and spatial scales of relevance to climate change. Indeed, understanding the major environmental problems of our future requires a combination of approaches and context‐dependent studies, where multiple sources of data might more reliably anticipate our future environment (Andersson et al., 2015; Boyd et al., 2018) in ways that are reproducible.

The search for reproducible results is a hallmark of science because it not only demonstrates knowledge on which we can rely but also provides confidence that a high degree of agreement can be reached by different researchers using different approaches (Nosek & Errington, 2020). Some fields of scientific research are currently suffering from a “reproducibility crisis” (Baker, 2016) and ecological research is particularly susceptible due to the combination of the complexity of ecosystems with logistic restrictions of field work that create situational‐dependency rather than generality (O'Grady, 2020). The reproducibility and repeatability of research on ocean acidification effects on fish behavior has recently been scrutinised (see Enserink, 2021 for a summary) with a focus on the behavior of a subset of tropical fish species. This attention has inadvertently put the credibility of ocean acidification research in the spotlight. Subsequent consideration has been to acknowledge that the outcomes of acidification assessments can be sensitive to testing conditions (Williamson et al., 2021) but emphasizing that ocean acidification is likely to contribute to the alteration of future ecological processes (Dupont et al., 2021; Nagelkerken et al., 2020).

If species community responses are indeed more dependent on predicted climate effects than investigation effects (i.e., testing conditions), these communities should show generalizable responses across samples (i.e., studies within a method), methods (i.e., field and laboratory methods), settings (i.e., different biogeographies and ecosystems), and taxa (i.e., different species within a functional group). Concerns over inappropriate experimental designs or replication (Cornwall & Hurd, 2016) or differences in experimental concentrations of CO_2_ and temperature should play a relatively smaller role if the influence of climate stressors is large. Similarly, this detectability should also remain where methods increasingly incorporate realism through use of complex mixtures of species (e.g., mesocosms), which are renown for accommodating plasticity of species interactions that can ramp‐up compensatory responses to dampen treatment effects (Goldenberg et al., 2018). Such effects should also hold in field conditions where ecological complexity is not only at its fullest but also potentially allows for multiple generations to respond (e.g., Leung et al., 2020).

To test such generalizability (and therefore also replicability) of climate effects across natural communities, we compare community‐level responses to future climate at natural volcanic CO_2_ vents across four global ecosystems (temperate kelp forests, temperate rocky reefs, coral reefs and seagrasses) and in the laboratory (acidification and warming in mesocosms). Our key interest was whether the communities of these iconic ecosystems can persist the effects of climate treatments without change in their community composition. We contrast this analysis with common measures of biodiversity that are typically based on changes in total species numbers or abundances rather than changes in the identity of individual species (Magurran, 2021). We reveal a weakness in the use of common metrics of biodiversity to identify replicable climate responses across natural systems and laboratory systems, but we show relative consistency in the sensitivity of community composition to ocean acidification (i.e., deviating in their structure from present‐day communities) across various approaches, taxa, locations, and ecosystems.

## 2 MATERIALS AND METHODS

### 2.1 Meta‐analysis database

Our literature search used Web of Science (Clarivate Analytics) and included studies up until August 2018. Search topics included the following: “ocean acidification” AND “seep* or vent* or gradient*” AND “communit* or assemblage* or diversity or species richness or colonisation or settlement or colonization”. These search terms covered ocean acidification research performed in the field at natural CO_2_ seeps, vents, or natural gradients. To allow analysis of diversity and community structure indices, we restricted the search to studies performed at the community or assemblage level, excluding all single‐species studies. We did not include the search terms “pH” or “CO_2_” or “carbon dioxide” because this returned a large number of deep‐sea hydrothermal vent studies with extreme pH conditions that did not reflect end of century ocean acidification projections. However, elevated CO_2_ studies performed in natural coastal systems have “ocean acidification” in their title, abstract, or keywords. The search returned 256 studies, which were supplemented with 8 studies from our own records (Figure S1). These 264 studies were screened to confirm that they had been performed in natural systems and included multiple species (i.e., communities), reducing the number of studies to 85. After screening the data availability of these studies (or their supplements), 58 studies were retained harboring abundance data of species communities in natural systems. These 58 studies provided 5637 unique treatment‐specific (control and vent) entries of individual‐taxon benthic coverage (*n* = 1657) or densities (*n* = 3980).

To identify community‐level studies done in a laboratory environment, testing effects of elevated CO_2_ and temperature, we searched the literature (up until August 2018) for the topics: “ocean acidification or CO_2_ or carbon dioxide or pH” AND “mesocosm*” AND “communit* or assemblage* or diversity or species richness or colonisation or settlement or colonization” AND “temperature or climate change or warming” AND “ocean* or sea* or marine”. We included the term “mesocosm” to disregard single‐species studies performed in small aquaria as our focus was community‐level studies. The search returned 166 studies, which were supplemented with 4 studies from our own records (Figure S1). These 170 studies were screened to confirm that they had been performed on multiple species, reducing the total number of studies to 43. After screening the data availability of these studies (or their supplements), 23 studies were retained harboring abundance data of species communities in laboratory systems subjected to elevated CO_2_ and/or temperature. These 23 studies provided 1513 unique treatment‐specific entries of individual‐taxon benthic coverage or densities.

### 2.2 Data retrieval and analysis

For each study we extracted the abundance (# individuals per experimental unit or per unit surface area) or the benthic cover (%) of each taxon within their respective communities from controls and their paired climate treatments (CO_2_ at vents; CO_2_, temperature and/or their combined effects from mesocosms). We used WebPlotDigitizer 4.2 (https://apps.automeris.io/wpd/) to retrieve data values from the respective graphs of the selected studies. Communities sampled for more than one season of the year were averaged across the year to produce a single observation (for both natural systems and mesocosms), whilst communities sampled at multiple sites were considered as replicates to produce replicate sites (not applicable for mesocosms).

We separated natural systems and mesocosm studies by study location, and for natural systems also by type of ecosystem. The latter was not possible for mesocosms as most of the studies did not reconstruct an ecosystem, but rather focussed on a selection of species often without introducing natural habitat. Natural systems were represented as one of four major biogenic habitat types: canopy reefs (temperate rocky reefs with a cover at control sites of >15% for the canopy‐forming fucoids *Cystoseira* spp. or for the kelp *Ecklonia radiata*), non‐canopy reefs (dominated by non‐canopy forming erect calcareous algae), coral reefs (dominated by tropical corals), and seagrass beds (dominated by seagrasses) and also retrieved data on total (biogenic) habitat cover of these habitat‐constructing species for each study. Although for seagrass beds, seagrass cover was quantified to evaluate degree of habitat change, the biodiversity and community metrics were predominantly calculated for the epibiont community of the seagrass leaves.

For all studies (natural and laboratory systems) we also retrieved data of the respective control and experimental levels of pH, *p*CO_2_, and/or temperature. For natural systems, the average change in seawater chemistry (control vs. CO_2_ vent, mean ± SD: ΔpH = −0.31 ± 0.16, Δ*p*CO_2_ = +656 ± 337 ppm) approximated a representative concentration pathway (RCP) of 8.5 greenhouse gas emission scenario for the year 2100 (Bopp et al., 2013). For mesocosms, the change in seawater chemistry (ΔpH = −0.25 ± 0.08, Δ*p*CO_2_ = +478 ± 124 ppm) ranged between an RCP 6.0 and 8.5 scenario, while the seawater temperature increase (+3.9 ± 1.2°C) was reflective of an RCP 8.5 scenario.

For each community of each respective climate treatment of each biogenic habitat, we calculated qualitative (based on species presence/absence) and quantitative (based on species abundances) measures of biodiversity and community change. Qualitative measures included taxonomic α‐diversity (species richness), β‐diversity, species turnover, species nestedness, species overlap, species lost, and species gained. Quantitative measures included evenness, community dissimilarity, and species dominance shifts.

Because species richness is very sensitive to sampling intensity and sampling surface area (which varied across studies) and because studies varied considerable in the total number of taxonomic groups sampled or included, we standardised α‐diversity by expressing species richness at control and vent sites as a relative abundance of the total local (i.e., site‐specific) species pool. Thus, for each study site we divided the number of taxa observed at controls (C1) and vents (C2), respectively, by the total number of taxa at site level (*a* + *b* + *c*) and multiplied this by 100% (see Venn diagram below):

$$\alpha ‐\text{diversity control}=\left(a+b\right)/\left(a+b+c\right)\times 100\%,$$

$$\alpha ‐\text{diversity vent}=\left(a+c\right)/\left(a+b+c\right)\times 100\%.$$

Taxonomic β‐diversity was calculated using Jaccard's dissimilarity index (Villéger et al., 2013):
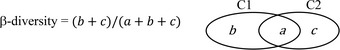
where *b* = number of taxa unique to community 1 (C1), *c* = number of taxa unique to community 2 (C2), and *a* = number of taxa shared by C1 and C2. β‐diversity equals 0 when C1 and C2 share the same species (*a* = C1 = C2) and equals 1 when they share no species (*a* = 0). However, β‐diversity can also approach 1 when some species are shared and when the number of species in one community is much higher than that in the other. Hence, β‐diversity is driven by differences in species turnover as well as differences in species richness. To separate these effects, β‐diversity can expressed as the sum of a turnover component (species replacement between the two communities; first part of the equation below) and a nestedness‐resultant component (affected by differences in species numbers between the two communities; second part of the equation), following Villéger et al. (2013):

$$\frac{b+c}{a+b+c}=\frac{2\times \min\left(b,c\right)}{a+2\times \min\left(b,c\right)}+\frac{\mathrm{abs}\left(b-c\right)}{a+b+c}\times \frac{a}{a+2\times \min\left(b,c\right)}.$$

Species turnover equals 0 when there is complete species overlap between the two communities, and equals 1 when the two communities share no species. Nestedness equals 0 when the two communities have the same number of species or have a different number of species with unique composition (*a* = 0), and tends to 1 when the smaller community hosts a small subset of the larger community and completely overlaps in composition with the larger community.

Comparable with species richness, species overlap (*a* in the Venn diagram), species lost at vents (*b*), and species gained at vents (*c*), were expressed as a percentage of the local (i.e., site‐specific) species pool (*a* + *b* + *c*; Avolio et al., 2019).

Quantitative measures of biodiversity change included community evenness, species dominance shifts (i.e., species rank re‐ordering), dissimilarity in community composition between controls and their paired vent site, and degree of heterogeneity within control and vent communities, respectively (i.e., similarity in community composition amongst all controls and amongst all vents, respectively). Because studies varied considerably in the total number of taxonomic groups that comprised the respective community, we used the Simpson (E_1/D_) index to calculate community evenness. This index is independent of species richness (and hence not dependent on differences in sampling effort among studies), ranges between 0 and 1 (making studies comparable on the same scale), and is sensitive to density variation of rare species (which are the taxa predicted to be sensitive to climate stress; Beisel et al., 2003).

Dissimilarity between control and their associated vent community was calculated using the Bray Curtis similarity index (dissimilarity = 100% − similarity), based on square‐root transformed data to de‐emphasise the contribution of highly abundant taxa to the analysis (following Kroeker, Gambi, et al., 2013). The dissimilarity was calculated for communities (i.e., species represented as individual entities) as well as for functional groups (i.e., species combined into functional groups—see paragraph below).

Community heterogeneity was calculated using the Bray Curtis similarity index with square‐root transformed data. For each of the four ecosystems, community similarity was calculated amongst all paired combinations of vent sites and among all paired combinations of control sites, respectively. This allowed us to compare the degree of global community homogenization under elevated CO_2_ conditions versus that at control conditions. For this analysis, however, community compositions could not be compared based on species‐level abundances because (1) the wide range of studies included were from across the world (i.e., different biogeographic zones and latitudinal distributions) and (2) many studies focussed on specific taxonomic groups, which precluded us from calculating a community similarity index across all studies (i.e., the species‐specific data among studies could not be fit into an single matrix due to lack of species overlap across biogeographic areas or missing data for certain taxonomic groups because they were not surveyed). Therefore, individual taxa were grouped into broader functional taxonomic groups that occurred at all locations. As a result, this analysis of community homogenization was performed for a reduced dataset of 52 communities (from the original 108 communities). The broader functional taxonomic groups distinguished (following Kroeker, Gambi, et al., 2013; Kroeker, Micheli, et al., 2013) were heavily calcified (calcareous) filter feeders (e.g., hard corals, bryozoans, benthic hydroids), non‐ or marginally‐calcified filter feeders (e.g., sponges, ascidians, anemones, soft corals), crustose coralline algae (encrusting calcareous algae), erect calcareous algae (fleshy algae with calcified structures or skeletons), turf/biofilm/ (e.g., fleshy turf algae, cyanobacteria, diatoms, microphytobenthos, filamentous algae), fleshy algae, and canopy‐forming vegetation (kelp, brown fucoids, seagrass). Studies that quantified the numbers or density of taxa (typically of animals) were excluded from this analysis as they spanned a much more diverse range of taxonomic groups than for the benthic cover data whilst many studies typically focussed on restricted selection of taxa. As a result we could not create a single matrix (i.e., with each taxa having a value for each location) for taxonomic groups for these studies that allowed comparison across locations from different biogeographies. The above functional analysis was not only used to compare spatial heterogeneity (i.e., within all controls and within all vents) but also to compare functional groups of controls with their associated vents.

Shifts in the dominance of species (rank shifts) were calculated following Avolio et al. (2019). First, all species were ranked separately for each control and their vent community, with species with the same cover or density within a community receiving the same rank. Then, for each species the difference between than rank at control versus vent was calculated. Then, these rank differences were averaged for the community and divided by the highest rank value to allow comparison among communities comprising different numbers of species.

For the mesocosm studies we used the same approach for the analyses as described for the natural systems above. However, the data could not be separated into unique biogenic habitats because most mesocosm studies did not recreate habitats. Species richness, species gained and species lost in each climate treatment was expressed as a percentage of the total number of taxa across all experimental treatments of each study (control, ocean acidification, ocean warming, acidification and warming combined). Species gained and species lost in each climate treatment was assessed by comparison of species occurrences at their respective controls. Community heterogeneity of functional taxonomic groups within treatments could not be evaluated among mesocosm studies, as they typically focussed on specific taxonomic groups (e.g., only invertebrates, fishes, corals, microbes, or phytoplankton) that precluded the capacity to compare across broader functional taxonomic entities.

### 2.3 Statistical analyses

We tested for differences in response of community evenness and relative species richness using ANOVAs based on a randomised block design (i.e., where locations were included as a random factor, but their interaction with other factors excluded due to lack of consistent replication within locations). For natural systems we included treatment (control vs. CO_2_ vents) as a fixed factor, taxonomic group as a random factor, and location as a random blocking factor. The four ecosystems were tested individually based on the a priori knowledge of their contrasting sensitivities to elevated CO_2_ (i.e., calcifying species typically decrease and weedy vegetation increase under CO_2_ enrichment; Nagelkerken & Connell, 2015; Sunday et al., 2017). For laboratory mesocosms, we included CO_2_ treatment (control vs. elevated) and temperature treatment (control vs. elevated) as fixed factors, taxonomic group as a random factor, and location as a random blocking factor. Data were not transformed (except for a fourth‐root transformation for evenness of canopy reefs), and a Monte Carlo test was used in case of low number of permutations. Traditional meta‐analysis based on an effect size (e.g., lnRR, Hedges' *d*) was not possible because studies did not report data from their lowest units of study (i.e., studies typically provided only mean values for community compositions at controls versus treatment).

In addition to comparing species richness and evenness among treatments (above), we also compared the number of studies that showed an increase versus a decrease versus no change in species richness or evenness at vents with ecosystems or within mesocosm treatments. For each study, we first determined whether the respective climate treatment showed a reduction of species richness, an increase in richness, or no change in richness, compared to their paired controls, respectively. Then, for each ecosystem and each mesocosm climate treatment we compared the number of positive, negative, and no change responses (“direction of change”, fixed factor) using a randomised block design with taxonomic group as a random factor, and location as a random blocking factor.

For species replacement, we performed a randomised block design with species change (natural systems: gains vs. losses at CO_2_ vents; mesocosms: gains vs. losses under ocean acidification, temperature, or acidification and temperature combined, respectively) as a fixed factor, taxonomic group as a random factor, and location as a random blocking factor.

The metrics of species overlap, community dissimilarity, *β*‐diversity, species nestedness, species turnover, and species dominance shift are all calculated by comparing a treatment (e.g., vent site or temperature in mesocosm) with its control, and as such return a single value (rather than a treatment and control value). Therefore, these metrics were best tested statistically among the four ecosystems and among the three mesocosm climate treatments. A randomized block design (ANOVA) was used for natural systems and mesocosms separately, testing the effect of system (sy: 4 ecosystems for in situ studies and 3 climate treatments for mesocosms, respectively), taxonomic group (ta; random factor, nested within system), and location (lo; random blocking factor).

To compare the similarity amongst, and explanatory power of the various biodiversity and community metrics, we performed a multivariate principal coordinates (PCOs) analysis based on an Euclidian distance matrix. The various biodiversity and community metrics were first standardised to fit along a common axis (“normalised” within PRIMER), and then drawn as vector onto the first two axes of the PCO plot. These vectors represent the Pearson correlations of the individual biodiversity and community metrics with the respective axes.

Differences in cover of habitat between controls and vents were tested on log_10_‐transformed data using a randomized block design (ANOVA), testing CO_2_ treatment (control vs. vent) as fixed factor and location as a random blocking factor. Seagrass cover was only available for one location and hence was tested with a one‐way ANOVA on log_10_/fourth root‐transformed data. Differences in composition of functional groups between controls and vents were tested using a randomized block design (ANOVA) with CO_2_ treatment (control vs. vent) and ecosystems (four types) as fixed factors, and location as a random blocking factor. This analysis was followed by a similarity percentages analysis (SIMPER) to calculate the contribution of each functional group to the differences in functional group composition between controls and vents. Community heterogeneity across controls communities and across vent communities (both based on functional groups rather than species) was tested for each ecosystem with a one‐way ANOVA on non‐transformed data with treatment (control vs. CO_2_ vent) as a fixed factor.

All ANOVAs above as well as the PCOs analysis were performed with the program PRIMER version 7. We used non‐parametric ANOVAs based on permutations rather than parametric ANOVAs because most of the biodiversity and community variables are not expressed on a ratio scale.

Simple linear regressions were performed for each ecosystem to test the relationship between: (1) the various biodiversity and community metrics and change in habitat cover, (2) the change in habitat cover and change in pH level at vents, and (3) the various biodiversity and community metrics and change in pH, *p*CO_2_ and temperature levels at vents, respectively. These tests were performed using IBM spss Statistics version 25.

The raw data used in the statistical analyses are publicly available from Nagelkerken and Connell (2022).

## 3 RESULTS

### 3.1 Reshuffling of functional groups at CO_2_ vents

**FIGURE 1:**
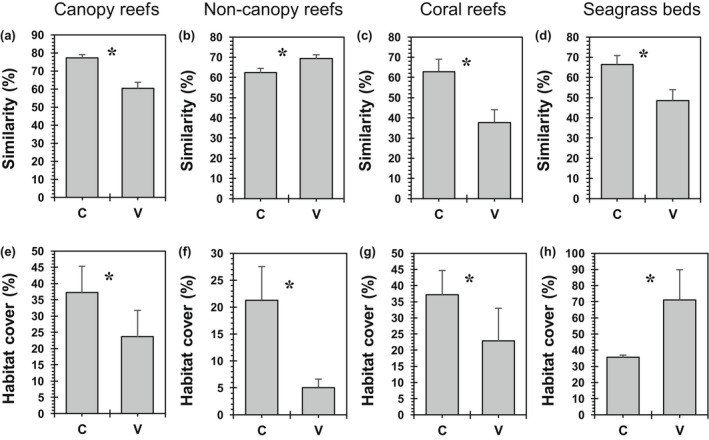
(a–d) Mean (+SE) similarity in functional group composition (Bray–Curtis) amongst all vent communities (V) and among all control communities (C) for each ecosystem. Species were subdivided into functional groups (see Section 2) to allow global comparison of communities with their unique species presences/absences. (e–h) Mean (+SE) biogenic habitat cover of major habitat builders at controls (C) and vents (V) for: (e) canopy reefs (habitat builders: fucoids and kelp), (f) non‐canopy reefs (erect calcareous algae), (g) coral reefs (hard corals), and (h) seagrass beds (seagrass plants). *Significant difference (see Table S1 for statistical details).

The composition of functional ecological groups differed between controls and natural CO_2_ vents (*p* = .0070), irrespective of differences amongst ecosystems (ecosystems *p* = .0001, treatment × ecosystem interaction *p* = .535, Table S1). A similar result was found when the analysis was extended to include studies that focussed only on algal functional groups (CO_2_ treatment *p* = .0021, ecosystem *p* = .0001, Table S1). Reshuffling of functional groups was predominantly caused by decreased cover of erect calcareous algae, crustose coralline algae, and calcified filter feeders but increased cover of fleshy algae, turf algae and biofilm at CO_2_ vents, respectively (SIMPER analysis, Table S1). Additionally, functional group composition became more heterogeneous across vent locations for canopy reefs, coral reefs and seagrass beds, respectively, but more homogeneous among non‐canopy reefs at vents (Figure 1; Table S1). Finally, in each of these ecosystems the cover of primary living habitat declined at vents compared with controls, except for seagrasses which increased at vents (Figure 1; Table S1).

### 3.2 Species replacement and community reshuffling under multiple climate stressors

**FIGURE 2:**
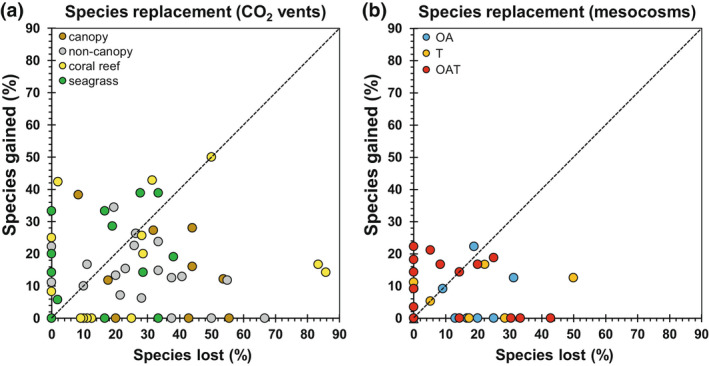
Percent species lost versus gained among (a) natural CO_2_ vents compared with their associated control sites and (b) mesocosm treatments: OA, ocean acidification; OAT, ocean warming and acidification; T, ocean warming. Each data point represents an individual study. The dotted line represents where data points would fall if there was an equal percentage of species lost and gained. Data points falling along the “0” of the *x*‐axis represent studies where species were only lost, while data points falling along the “0” of the *y*‐axis represent studies where species were only gained. Data points falling on the origin (0, 0) represent studies where no change in species numbers was observed. All other data points represent studies with species gains as well as losses. Statistical results comparing species gains versus losses are shown in Table S2, while graphs showing species gains versus losses for individual taxonomic groups are shown in Figure S2.

Ecological responses to climate stress at taxonomic levels was best characterised by “species replacements” (gains and losses in species) and “community reshuffling” (change in abundance or benthic cover of species). Species replacements occurred for 59% of the studies in natural systems versus 49% in mesocosms (Figure 2). The mean number of species lost did not differ significantly from that of mean number of species gained for the natural systems (non‐canopy reef: 14 vs. 6; coral reef: 11 vs. 8; seagrass: 12 vs. 17, respectively) and mesocosm treatments (OA: 7 vs. 3; T: 7 vs. 4; OAT: 7 vs. 6), except for canopy reefs (35 vs. 10), which had higher mean numbers of species lost than gained (Figure S2; *p* = .0298; Table S2).

Community reshuffling was more prominent in natural systems (99% of studies) than for mesocosms (76%). On average, community reshuffling caused a 29%–37% dissimilarity in composition between control and vent communities across ecosystems, and a 17%–22% dissimilarity for mesocosm studies across climate treatments (see Figure S7e).

In natural systems, individual canopy reefs showed either an increase or decrease in kelp canopy cover at vents (although averaged across all studies canopy cover was lost, Figure 1e), yet species of turf algae and fleshy algae also emerged as dominant vegetation types (Figure S3), increasing community heterogeneity at vents (see Figure 1a–d). On non‐canopy reefs, community heterogeneity decreased as benthic cover of species with calcified skeletons (i.e., erect calcareous algae, crustose coralline algae, calcified filter feeders) largely disappeared as dominant vegetation types at vents and were consistently replaced by species of turf algae, fleshy algae, and biofilms as dominants across vents. On coral reefs, increased community heterogeneity was driven by reduced cover of hard coral and crustose coralline algae as dominant species and increased cover of species of turfs, fleshy algae, biofilm, soft corals, or sponges as dominants. Likewise, community heterogeneity increased in seagrass beds where crustose coralline algae as the dominant epiphyte species on seagrasses at controls were replaced by increased cover of alternative taxa, that is, bryozoans, turfs, or erect calcareous algae at vents, while seagrass cover often increased. Species of amphipods and foraminifera generally increased in abundance at CO_2_ vents (Figure S4), while species abundances of hard corals, barnacles, echinoderms, and isopods consistently declined at vents, with the remaining taxa showing mixed responses of increases and decreases at vents.

In mesocosms, species of amphipods, copepods, fishes, turf, and zooplankton often increased across climate treatments (Figure S5).

### 3.3 Biodiversity change under multiple climate stressors

**FIGURE 3:**
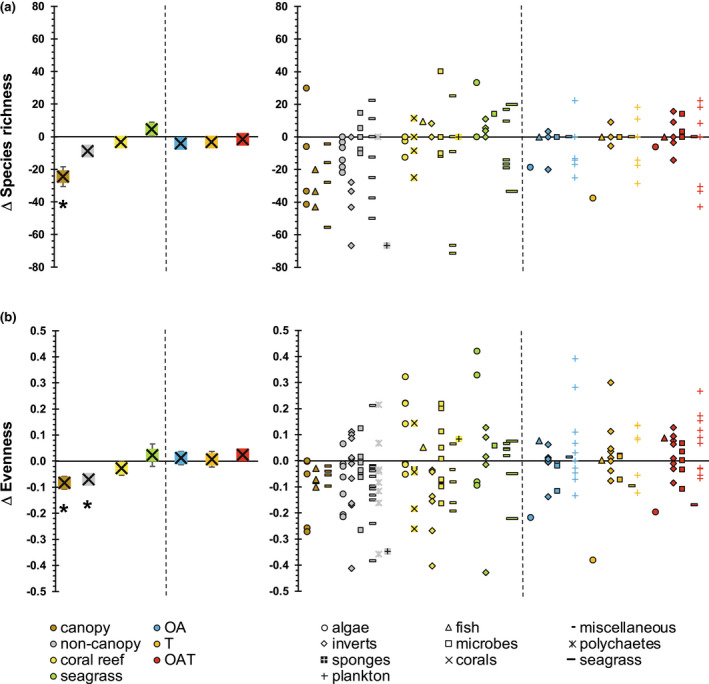
Mean (±SE; left‐hand panels) (a) change in species richness, and (b) change in community evenness, between control sites and their associated CO_2_ vent sites for each of the four ecosystems, and between control mesocosms and their associated mesocosm treatments. OA, ocean acidification; OAT, ocean warming and acidification; T, ocean warming. *Indicates a significant difference between control and vents for individual ecosystems; detailed statistical results are shown in Table S3a. The left‐hand graphs shows the difference (i.e., relative change) in species richness/evenness between controls and climate treatments (Δ); absolute mean values of species richness and evenness are shown in Figure S6. Data points in right‐hand panels represent individual studies and their taxa studied.

Mean species richness (i.e., number of species) at CO_2_ vents was reduced for temperate canopy (kelp) reefs only (Figures 3; Figure S6; *p* = .0326; Table S3a), while mean species evenness (i.e., how even the abundance distribution of species is across the community) was reduced at CO_2_ vents for temperate canopy‐ and non‐canopy reefs only (*p* = .0170 and .0403, respectively). These patterns occurred independently of the detected effects of taxon or location. In contrast, no CO_2_ or temperature effects were detected on species richness or evenness for the other vent ecosystems (corals, seagrasses) or any mesocosm study (Figure S6; Table S3a). This lack of detectable change was the result of similar numbers of studies observing a decrease versus an increase in species richness or evenness (Figure 3; Table S3b), except for evenness of invertebrates on coral reefs and for richness of algal and invertebrate species on non‐canopy reefs, which showed a higher incidence of decrease than increase.

Other metrics of biodiversity change (species overlap, species turnover, species nestedness, *β*‐diversity, community dissimilarity, and species dominance shifts) did not differ among the three mesocosm climate treatments, but species overlap, species turnover, and species nestedness of canopy and non‐canopy reefs differed from that of coral reefs, with canopy reefs showing the most deviating mean values (Figure S7; Table S4).

Multivariate analysis of the various biodiversity metrics showed that differences between natural systems and mesocosms were best explained by species overlap, species gained, species turnover, *β*‐diversity, community dissimilarity, and dominance shifts, with the first three metrics showing the strongest correlations (Figure S8).

### 3.4 Correlations of biodiversity change with habitat loss and magnitude of climate stress

There was no significant relationship between extent of habitat loss and various biodiversity and community change metrics, except for between habitat loss and community dissimilarity at canopy reefs (*R* ^2^ = .62, *p* = .020; Table S5). The extent of habitat loss was not related to the level of pH reduction at the vent sites (*R* ^2^ < .17, *p* ≥ .361; Table S5). Moreover, none of the biodiversity and community change metrics detected either a significant or a notable relationship (*R* ^2^ range .03–.13 for significant relationships) with magnitude of pH decline, *p*CO_2_ increase, or temperature increase (Table S5).

## 4 DISCUSSION

Pioneering research on ocean acidification predicted the loss of calcifiers (Fabry et al., 2008; Orr et al., 2005), and homogenization of habitats to create ecosystems of simplified functions (Kroeker, Micheli, et al., 2013). Across the globe there has been substantial research investment into assessing the susceptibility of individual species and communities to anticipate these future ecosystems (Kroeker et al., 2010; Nagelkerken & Connell, 2015), but clear predictions have not always emerged across the range of techniques used (Andersson et al., 2015; Wernberg et al., 2012). We consider that this lack of clarity reflects the context‐dependency with which ocean acidification may drive ecosystem change. Future changes to marine biodiversity appear less consistent or widespread than forecast by some studies, which were often focussed on species most sensitive to reduced pH, notably calcifying species (Leung et al., 2022). We found that the strongest influence of ocean acidification occurred within natural communities at volcanic CO_2_ vents. Whilst the study of any one locality often showed either a gain or loss in total species richness and evenness, which collectively cancelled directional change at a global scale, observations of the majority of vents reveal global‐scale divergence of community compositions from current day patterns. Our comparison of communities amongst vents also shows many of them to be spatially more heterogeneous than the global set of communities that represent present‐day conditions. These findings underscore the mediating role of ocean acidification as a future driver of community change across ecosystems globally.

A striking feature of our global analysis is the propensity for acidification to drive replacements of species across a wide range of taxa and functional groups, rather than net losses or gains of species. Unsurprisingly, it is those metrics that calculate species replacement (e.g., species gains and losses) and community reshuffling (e.g., dissimilarity in communities and functional composition, and spatial community heterogeneity) that tended to be more sensitive in our analysis in detecting community change, because they incorporate the identity of taxa being gained or lost as well as changes in their relative abundances (Bastazini et al., 2021; Hillebrand et al., 2018). This “reshuffling” of species occurrence and abundance was more consistently observed (and therefore more reproducible) than the straight‐forward loss of species per se. Moreover, because species tended to be lost at similar rates as they were gained across studies, metrics based on average change in numbers of species (e.g., species richness—also referred to as *α*‐diversity) that do not incorporate species identity were generally insensitive in detecting persistent patterns. This finding reinforces the concept that climate change can act as a resource as well as a stressor to species (Connell et al., 2018), with ensuing community reorganization rather than persistent species loss (e.g., as also found in plant communities, Komatsu et al., 2019). Nevertheless, it is the type of species, and in particular their functional roles which are lost that matters. If lost species are replaced with species performing different functional roles, ecosystem functional diversity and as a consequence ecosystem function and stability can be lost (Kroeker, Micheli, et al., 2013; Teixido et al., 2018). Loss of habitat‐forming species (e.g., corals, kelp) can further have cascading effects on associated species and functional diversity (Graham et al., 2006). Additionally, loss or gain of animal and algal species or change in their abundances can directly affect their consumers (Ghedini & Connell, 2016), leading to bottom‐up driven alterations to food web structure, stability, and energy flow (Nagelkerken et al., 2020). In sum, our quantitative global approach reveals the power of combining disparate studies across taxa, locations, methods and biogeographies to detect prevailing and often reproducible observations of global community reshuffling under ocean acidification.

Mesocosm studies in general showed lower sensitivity in detecting species replacements, community reshuffling or biodiversity change than natural systems, either to ocean acidification, warming or their combined effect. This lower‐sensitivity is not unexpected as community structure is often mediated by ecological processes that operate over substantially larger spatial and temporal scales than those tested in mesocosms. For example, most mesocosm studies are of short duration (weeks to months) and therefore cannot fully encapsulate the longer‐term ecological processes that drive species replacements in nature (e.g., growth, mortality, disease, reproduction). Indeed, a long‐term (2‐year) mesocosm study on coral reef cryptobenthos did observe species reshuffling under the combined effect of ocean acidification and warming in the absence of species diversity loss (Timmers et al., 2021), with a similar finding for a temperate benthic community under ocean acidification after a half‐year exposure in mesocosms (Nagelkerken et al., 2020). Furthermore, natural processes such as allochthonous larval replenishment or animal migrations can buffer local diversity loss or population declines in nature under climate stress, but these processes are largely absent in mesocosms, or substantially constrained. In addition, the number of species is non‐linearly related to sampling area (Connor & McCoy, 1979) and mediated by spatial heterogeneity (McGill et al., 2015). Therefore, due to the restricted size of mesocosms, their biodiversity responses are more sensitive to specific choice of focal species and microhabitats compared with natural systems, and therefore tend to reflect the specificity of mesocosm collections and conditions. Hence, our finding that biodiversity and community change responses are more readily observed in natural systems exposed to ocean acidification, reinforces the idea that meta‐population dynamics (e.g., Brustolin et al., 2019) and ecological complexity (e.g., Goldenberg et al., 2018) are strong mediators of species responses to climate change, and hence form a critical component in assessments of reproducibility of ecosystem responses to climate stress.

Species diversity is often mediated by the amount and complexity of habitat (Graham et al., 2006), but we observed no consistent relationship between habitat loss and change in species diversity or community structure in natural systems. An earlier meta‐analysis showed that ocean acidification reduces structural complexity of coral reefs and mussels beds (Sunday et al., 2017). Based on the negative relationship between habitat structural complexity and biodiversity, it was predicted that habitat loss due to ocean acidification would consequently result in loss of biodiversity in these two major habitats (Sunday et al., 2017). Our quantitative analysis only found an incidental relationship between habitat loss and diversity, that is, only for canopy reefs, but not for the other major habitats. This apparent contradiction might be explained by our observation that species tended to be lost at similar rates as they were gained under ocean acidification, with sensitive species likely being replaced by more resilient species. Such replacement species are often opportunistic species that prevail in disturbed environments such as under climate stress (McKinney & Lockwood, 1999), are physiologically less sensitive to pH‐reduction (Wittmann & Portner, 2013), or able to genetically adapt to elevated CO_2_ (Sunday et al., 2014). Moreover, loss of biogenic habitat can create new space for smaller organisms to colonise and increase habitat heterogeneity, increasing rather than decreasing biodiversity (Tews et al., 2004). Although habitat loss is still one of the major drivers of marine biodiversity loss globally (Lotze, 2021), loss of major biogenic habitats due to ocean acidification can also facilitate the emergence of alternate species. However, this might not be beneficial per se as it is the ecological functions that species of these novel communities perform that matter for the maintenance of ecosystem functioning under climate change, rather than species diversity (Maureaud et al., 2019; Teixido et al., 2018). Indeed, we observed that the functional composition of communities diverged spatially across vents, and also differed from that of their associated control sites. This strengthens our assertion that species identity matters in forecasting the effects of ocean acidification on our future oceans.

We conclude that species replacements and community reshuffling under ocean acidification can occur in the absence of wholesale loss of species diversity and can be mediated by loss of major biogenic habitat (i.e., providing opportunities for other species to emerge), being much more obvious in natural systems than in laboratory mesocosms. Species replacement and community reshuffling under elevated CO_2_ represent critical processes that appear largely reproducible in studies across the globe, irrespective of study‐specific differences across taxa, ecosystems, and locations. Because of these persistent influences of ocean acidification, future projections of ecosystem change and stability will be more meaningful if they focus on detections of species replacements and changes to their abundances rather than test for signs of habitat loss or biodiversity loss per se. Although this influence of species identity is less noticeable than their loss, such changes can drive the dynamics of ecosystem stability or functional change.

## FUNDING INFORMATION

I.N. was supported by an Australian Research Council Future fellowship (grant no. FT120100183), while S.D.C. was supported by an ARC Linkage grant (grant no. LP200201000).

## CONFLICT OF INTEREST

The authors declare no competing interests.

## Supporting information


Appendix S1.

## Data Availability

The data that support the findings of this study are openly available in Dryad at https://doi.org/10.5061/dryad.98sf7m0mv.
