# Supplementary material for: Ocean acidification drives global reshuffling of ecological communities
Source: Glob Chang Biol. 2022 Sep 29;28(23):7038–48. doi: 10.1111/gcb.16410 (PMC9828364; doi:10.1111/gcb.16410)
Supplement: Supplementary file 1 — Appendix S1. [file GCB-28-7038-s001.docx]

**Supporting Information**

**Ocean acidification drives global reshuffling of ecological communities**

**Ivan Nagelkerken and Sean D. Connell**

**Fig. S1a** PRISMA flow diagram (obtained from Moher et al. 2009) for data search on effects of elevated CO_2_ on species communities in natural ecosystems using Web of Science (up until Aug. 2018). Search terms were TOPIC: (ocean acidification) AND TOPIC: (seep* or vent* or gradient*) AND TOPIC: (communit* or assemblage* or diversity or species richness or colonisation or settlement or colonization). References of all included studies are listed at the end of the flow diagram.

Studies included in qualitative synthesis
(n = 58)

Full-text articles excluded, with reasons
(n = 27)

Full-text articles assessed for eligibility
(n = 85)

Records excluded
(n = 179)

Records screened
(n = 264)

Records after duplicates removed
(n = 264)

## Identification

## Eligibility

## Included

## Screening

Studies included in quantitative synthesis
(n = 58 studies, n = 228 communities, n = 5,637 unique species × treatment entries)

Additional records identified through other sources
(n = 8)

Records identified through database searching
(n = 256)

Agostini, S., Harvey, B. P., Wada, S., Kon, K., Milazzo, M., Inaba, K., & Hall-Spencer, J. M. (2018). Ocean acidification drives community shifts towards simplified non-calcified habitats in a subtropical−temperate transition zone. *Scientific Reports, 8*(1), 11354. doi:10.1038/s41598-018-29251-7

Allen, R., Foggo, A., Fabricius, K., Balistreri, A., & Hall-Spencer, J. M. (2017). Tropical CO2 seeps reveal the impact of ocean acidification on coral reef invertebrate recruitment. *Marine Pollution Bulletin, 124*(2), 607-613. doi:10.1016/j.marpolbul.2016.12.031

Baggini, C., Issaris, Y., Salomidi, M., & Hall-Spencer, J. (2015). Herbivore diversity improves benthic community resilience to ocean acidification. *Journal of Experimental Marine Biology and Ecology, 469*, 98-104. doi:10.1016/j.jembe.2015.04.019

Baggini, C., Salomidi, M., Voutsinas, E., Bray, L., Krasakopoulou, E., & Hall-Spencer, J. M. (2014). Seasonality Affects Macroalgal Community Response to Increases in pCO(2). *Plos One, 9*(9). doi:10.1371/journal.pone.0106520

Bellissimo, G., Lluch, J. R., Tomasello, A., & Calvo, S. (2014). The community of Cystoseira brachycarpa J. Agardh emend. Giaccone ( Fucales, Phaeophyceae) in a shallow hydrothermal vent area of the Aeolian Islands ( Tyrrhenian Sea, Italy). *Plant Biosystems, 148*(1), 21-26. doi:10.1080/11263504.2013.778350

Brown, N. E. M., Milazzo, M., Rastrick, S. P. S., Hall-Spencer, J. M., Therriault, T. W., & Harley, C. D. G. (2018). Natural acidification changes the timing and rate of succession, alters community structure, and increases homogeneity in marine biofouling communities. *Global Change Biology, 24*(1), E112-E127. doi:10.1111/gcb.13856

Cattano, C., Calo, A., Di Franco, A., Firmamento, R., Quattrocchi, F., Sdiri, K., . . . Milazzo, M. (2017). Ocean acidification does not impair predator recognition but increases juvenile growth in a temperate wrasse off CO2 seeps. *Marine Environmental Research, 132*, 33-40. doi:10.1016/j.marenvres.2017.10.013

Chan, B. K. K., Wang, T. W., Chen, P. C., Lin, C. W., Chan, T. Y., & Tsang, L. M. (2016). Community Structure of Macrobiota and Environmental Parameters in Shallow Water Hydrothermal Vents off Kueishan Island, Taiwan. *Plos One, 11*(2). doi:10.1371/journal.pone.0148675

Cigliano, M., Gambi, M. C., Rodolfo-Metalpa, R., Patti, F. P., & Hall-Spencer, J. M. (2010). Effects of ocean acidification on invertebrate settlement at volcanic CO2 vents. *Marine Biology, 157*(11), 2489-2502. doi:10.1007/s00227-010-1513-6

Cornwall, C. E., Revill, A. T., Hall-Spencer, J. M., Milazzo, M., Raven, J. A., & Hurd, C. L. (2017). Inorganic carbon physiology underpins macroalgal responses to elevated CO2. *Scientific Reports, 7*. doi:10.1038/srep46297

Crook, E. D., Kroeker, K. J., Potts, D. C., Rebolledo-Vieyra, M., Hernandez-Terrones, L. M., & Paytan, A. (2016). Recruitment and Succession in a Tropical Benthic Community in Response to In-Situ Ocean Acidification. *Plos One, 11*(1), 16. doi:10.1371/journal.pone.0146707

Crook, E. D., Potts, D., Rebolledo-Vieyra, M., Hernandez, L., & Paytan, A. (2012). Calcifying coral abundance near low-pH springs: implications for future ocean acidification. *Coral Reefs, 31*(1), 239-245. doi:10.1007/s00338-011-0839-y

Donnarumma, L., Lombardi, C., Cocito, S., & Gambi, M. C. (2014). Settlement pattern of Posidonia oceanica epibionts along a gradient of ocean acidification: an approach with mimics. *Mediterranean Marine Science, 15*(3), 498-509. doi:10.12681/mms.677

Enochs, I. C., Manzello, D. P., Donham, E. M., Kolodziej, G., Okano, R., Johnston, L., . . . Price, N. N. (2015). Shift from coral to macroalgae dominance on a volcanically acidified reef. *Nature Climate Change, 5*(12), 1083-+. doi:10.1038/nclimate2758

Fabricius, K. E., De'ath, G., Noonan, S., & Uthicke, S. (2014). Ecological effects of ocean acidification and habitat complexity on reef-associated macroinvertebrate communities. *Proceedings of the Royal Society B-Biological Sciences, 281*(1775). doi:10.1098/rspb.2013.2479

Fabricius, K. E., Kluibenschedl, A., Harrington, L., Noonan, S., & De'ath, G. (2015). In situ changes of tropical crustose coralline algae along carbon dioxide gradients. *Scientific Reports, 5*. doi:10.1038/srep09537

Fabricius, K. E., Langdon, C., Uthicke, S., Humphrey, C., Noonan, S., De'ath, G., . . . Lough, J. M. (2011). Losers and winners in coral reefs acclimatized to elevated carbon dioxide concentrations. *Nature Climate Change, 1*(3), 165-169. doi:10.1038/nclimate1122

Fabricius, K. E., Noonan, S. H. C., Abrego, D., Harrington, L., & De'ath, G. (2017). Low recruitment due to altered settlement substrata as primary constraint for coral communities under ocean acidification. *Proceedings of the Royal Society B-Biological Sciences, 284*(1862). doi:10.1098/rspb.2017.1536

Ferreira, C. M., Nagelkerken, I., Goldenberg, S. U., & Connell, S. D. (2018). CO2 emissions boost the benefits of crop production by farming damselfish. *Nature Ecology & Evolution, 2*(8), 1223-1226. doi:10.1038/s41559-018-0607-2

Gambi, M. C., Musco, L., Giangrande, A., Badalamenti, F., Micheli, F., & Kroeker, K. J. (2016). Distribution and functional traits of polychaetes in a CO2 vent system: winners and losers among closely related species. *Marine Ecology Progress Series, 550*, 121-134. doi:10.3354/meps11727

Garrard, S. L., Gambi, M. C., Scipione, M. B., Patti, F. P., Lorenti, M., Zupo, V., . . . Buia, M. C. (2014). Indirect effects may buffer negative responses of seagrass invertebrate communities to ocean acidification. *Journal of Experimental Marine Biology and Ecology, 461*, 31-38. doi:10.1016/j.jembe.2014.07.011

Goodwin, C., Rodolfo-Metalpa, R., Picton, B., & Hall-Spencer, J. M. (2014). Effects of ocean acidification on sponge communities. *Marine Ecology-an Evolutionary Perspective, 35*, 41-49. doi:10.1111/maec.12093

Guilini, K., Weber, M., de Beer, D., Schneider, M., Molari, M., Lott, C., . . . Vanreusel, A. (2017). Response of Posidonia oceanica seagrass and its epibiont communities to ocean acidification. *Plos One, 12*(8). doi:10.1371/journal.pone.0181531

Hall-Spencer, J. M., Rodolfo-Metalpa, R., Martin, S., Ransome, E., Fine, M., Turner, S. M., . . . Buia, M. C. (2008). Volcanic carbon dioxide vents show ecosystem effects of ocean acidification. *Nature, 454*(7200), 96-99. doi:10.1038/nature07051

Hassenruck, C., Fink, A., Lichtschlag, A., Tegetmeyer, H. E., de Beer, D., & Ramette, A. (2016). Quantification of the effects of ocean acidification on sediment microbial communities in the environment: the importance of ecosystem approaches. *Fems Microbiology Ecology, 92*(5). doi:10.1093/femsec/fiw027

Hassenruck, C., Hofmann, L. C., Bischof, K., & Ramette, A. (2015). Seagrass biofilm communities at a naturally CO2-rich vent. *Environmental Microbiology Reports, 7*(3), 516-525. doi:10.1111/1758-2229.12282

Hassenruck, C., Tegetmeyer, H. E., Ramette, A., & Fabricius, K. E. (2017). Minor impacts of reduced pH on bacterial biofilms on settlement tiles along natural pH gradients at two CO2 seeps in Papua New Guinea. *Ices Journal of Marine Science, 74*(4), 978-987. doi:10.1093/icesjms/fsw204

Inoue, S., Kayanne, H., Yamamoto, S., & Kurihara, H. (2013). Spatial community shift from hard to soft corals in acidified water. *Nature Climate Change, 3*(7), 683-687. doi:10.1038/nclimate1855

Januar, H. I., Zamani, N. P., Soedarma, D., Chasanah, E., & Wright, A. D. (2017). Tropical coral reef coral patterns in Indonesian shallow water areas close to underwater volcanic vents at Minahasa Seashore, and Mahengetang and Gunung Api Islands. *Marine Ecology-an Evolutionary Perspective, 38*(2). doi:10.1111/maec.12415

Johnson, V., Brownlee, C., Milazzo, M., & Hall-Spencer, J. (2015). Marine Microphytobenthic Assemblage Shift along a Natural Shallow-Water CO2 Gradient Subjected to Multiple Environmental Stressors. *Journal of Marine Science and Engineering, 3*(4), 1425.

Johnson, V. R., Brownlee, C., Rickaby, R. E. M., Graziano, M., Milazzo, M., & Hall-Spencer, J. M. (2013). Responses of marine benthic microalgae to elevated CO2. *Marine Biology, 160*(8), 1813-1824. doi:10.1007/s00227-011-1840-2

Johnson, V. R., Russell, B. D., Fabricius, K. E., Brownlee, C., & Hall-Spencer, J. M. (2012). Temperate and tropical brown macroalgae thrive, despite decalcification, along natural CO2 gradients. *Global Change Biology, 18*(9), 2792-2803. doi:10.1111/j.1365-2486.2012.02716.x

Kerfahi, D., Hall-Spencer, J. M., Tripathi, B. M., Milazzo, M., Lee, J., & Adams, J. M. (2014). Shallow Water Marine Sediment Bacterial Community Shifts Along a Natural CO2 Gradient in the Mediterranean Sea Off Vulcano, Italy. *Microbial Ecology, 67*(4), 819-828. doi:10.1007/s00248-014-0368-7

Kroeker, K. J., Gambi, M. C., & Micheli, F. (2013). Community dynamics and ecosystem simplification in a high-CO2 ocean. *Proceedings of the National Academy of Sciences of the United States of America, 110*(31), 12721-12726. doi:10.1073/pnas.1216464110

Kroeker, K. J., Micheli, F., & Gambi, M. C. (2013). Ocean acidification causes ecosystem shifts via altered competitive interactions. *Nature Climate Change, 3*(2), 156-159. doi:10.1038/nclimate1680

Kroeker, K. J., Micheli, F., Gambi, M. C., & Martz, T. R. (2011). Divergent ecosystem responses within a benthic marine community to ocean acidification. *Proceedings of the National Academy of Sciences of the United States of America, 108*(35), 14515-14520. doi:10.1073/pnas.1107789108

Martin, S., Rodolfo-Metalpa, R., Ransome, E., Rowley, S., Buia, M. C., Gattuso, J. P., & Hall-Spencer, J. (2008). Effects of naturally acidified seawater on seagrass calcareous epibionts. *Biology Letters, 4*(6), 689-692. doi:10.1098/rsbl.2008.0412

Meron, D., Rodolfo-Metalpa, R., Cunning, R., Baker, A. C., Fine, M., & Banin, E. (2012). Changes in coral microbial communities in response to a natural pH gradient. *Isme Journal, 6*(9), 1775-1785. doi:10.1038/ismej.2012.19

Morrow, K. M., Bourne, D. G., Humphrey, C., Botte, E. S., Laffy, P., Zaneveld, J., . . . Webster, N. S. (2015). Natural volcanic CO2 seeps reveal future trajectories for host-microbial associations in corals and sponges. *Isme Journal, 9*(4), 894-908. doi:10.1038/ismej.2014.188

Muller, E. M., Fine, M., & Ritchie, K. B. (2016). The stable microbiome of inter and sub-tidal anemone species under increasing pCO(2). *Scientific Reports, 6*. doi:10.1038/srep37387

Munday, P. L., Cheal, A. J., Dixson, D. L., Rummer, J. L., & Fabricius, K. E. (2014). Behavioural impairment in reef fishes caused by ocean acidification at CO2 seeps. *Nature Climate Change, 4*(6), 487-492. doi:10.1038/nclimate2195

Nagelkerken, I., Goldenberg, S. U., Coni, E. O. C., & Connell, S. D. (2018). Microhabitat change alters abundances of competing species and decreases species richness under ocean acidification. *The Science of the total environment, 645*, 615-622. doi:10.1016/j.scitotenv.2018.07.168

Nagelkerken, I., Goldenberg, S. U., Ferreira, C. M., Russell, B. D., & Connell, S. D. (2017). Species Interactions Drive Fish Biodiversity Loss in a High-CO2 World. *Current Biology, 27*(14), 2177-+. doi:10.1016/j.cub.2017.06.023

Nagelkerken, I., Russell, B. D., Gillanders, B. M., & Connell, S. D. (2016). Ocean acidification alters fish populations indirectly through habitat modification. *Nature Climate Change, 6*(1), 89-+. doi:10.1038/nclimate2757

Nogueira, P., Gambi, M. C., Vizzini, S., Califano, G., Tavares, A. M., Santos, R., & Martinez-Crego, B. (2017). Altered epiphyte community and sea urchin diet in Posidonia oceanica meadows in the vicinity of volcanic CO2 vents. *Marine Environmental Research, 127*, 102-111. doi:10.1016/j.marenvres.2017.04.002

Pettit, L. R., Hart, M. B., Medina-Sanchez, A. N., Smart, C. W., Rodolfo-Metalpa, R., Hall-Spencer, J. M., & Prol-Ledesma, R. M. (2013). Benthic foraminifera show some resilience to ocean acidification in the northern Gulf of California, Mexico. *Marine Pollution Bulletin, 73*(2), 452-462. doi:10.1016/j.marpolbul.2013.02.011

Porzio, L., Buia, M. C., & Hall-Spencer, J. M. (2011). Effects of ocean acidification on macroalgal communities. *Journal of Experimental Marine Biology and Ecology, 400*(1-2), 278-287. doi:10.1016/j.jembe.2011.02.011

Porzio, L., Garrard, S. L., & Buia, M. C. (2013). The effect of ocean acidification on early algal colonization stages at natural CO2 vents. *Marine Biology, 160*(8), 2247-2259. doi:10.1007/s00227-013-2251-3

Raulf, F. F., Fabricius, K., Uthicke, S., de Beer, D., Abed, R. M. M., & Ramette, A. (2015). Changes in microbial communities in coastal sediments along natural CO2 gradients at a volcanic vent in Papua New Guinea. *Environmental Microbiology, 17*(10), 3678-3691. doi:10.1111/1462-2920.12729

Ravaglioli, C., Lauritano, C., Buia, M. C., Balestri, E., Capocchi, A., Fontanini, D., . . . Bulleri, F. (2017). Nutrient Loading Fosters Seagrass Productivity Under Ocean Acidification. *Scientific Reports, 7*. doi:10.1038/s41598-017-14075-8

Ricevuto, E., Kroeker, K. J., Ferrigno, F., Micheli, F., & Gambi, M. C. (2014). Spatio-temporal variability of polychaete colonization at volcanic CO2 vents indicates high tolerance to ocean acidification. *Marine Biology, 161*(12), 2909-2919. doi:10.1007/s00227-014-2555-y

Ricevuto, E., Lorenti, M., Patti, F. P., Scipione, M. B., & Gambi, M. C. (2012). TEMPORAL TRENDS OF BENTHIC INVERTEBRATE SETTLEMENT ALONG A GRADIENT OF OCEAN ACIDIFICATION AT NATURAL CO2 VENTS (TYRRHENIAN SEA). *Biologia Marina Mediterranea, 19*(1), 49-52.

Sangil, C., Clemente, S., Brito, A., Rodriguez, A., Balsalobre, M., Mendoza, J. C., . . . Hernandez, J. C. (2016). Seaweed community response to a massive CO2 input. *Estuarine Coastal and Shelf Science, 178*, 48-57. doi:10.1016/j.ecss.2016.05.025

Suggett, D. J., Hall-Spencer, J. M., Rodolfo-Metalpa, R., Boatman, T. G., Payton, R., Pettay, D. T., . . . Lawson, T. (2012). Sea anemones may thrive in a high CO2 world. *Global Change Biology, 18*(10), 3015-3025. doi:10.1111/j.1365-2486.2012.02767.x

Takahashi, M., Noonan, S. H. C., Fabricius, K. E., & Collier, C. J. (2016). The effects of long-term in situ CO2 enrichment on tropical seagrass communities at volcanic vents. *Ices Journal of Marine Science, 73*(3), 876-886. doi:10.1093/icesjms/fsv157

Taylor, J. D., Ellis, R., Milazzo, M., Hall-Spencer, J. M., & Cunliffe, M. (2014). Intertidal epilithic bacteria diversity changes along a naturally occurring carbon dioxide and pH gradient. *Fems Microbiology Ecology, 89*(3), 670-678. doi:10.1111/1574-6941.12368

Triantaphyllou, M. V., Baumann, K. H., Karatsolis, B. T., Dimiza, M. D., Psarra, S., Skampa, E., . . . Nomikou, P. (2018). Coccolithophore community response along a natural CO2 gradient off Methana (SW Saronikos Gulf, Greece, NE Mediterranean). *Plos One, 13*(7), 21. doi:10.1371/journal.pone.0200012

Vizzini, S., Martinez-Crego, B., Andolina, C., Massa-Gallucci, A., Connell, S. D., & Gambi, M. C. (2017). Ocean acidification as a driver of community simplification via the collapse of higher-order and rise of lower-order consumers. *Scientific Reports, 7*. doi:10.1038/s41598-017-03802-w

**Fig. S1b** PRISMA flow diagram (obtained from Moher et al. 2009) for data search on effects of elevated CO_2_ on species communities in laboratory mesocosms using Web of Science (up until Aug. 2018). Search terms were TOPIC: (ocean acidification or CO_2_ or carbon dioxide or pH) AND TOPIC: (mesocosm*) AND TOPIC: (communit* or assemblage* or diversity or species richness or colonisation or settlement or colonization) AND TOPIC: (temperature or climate change or warming) AND TOPIC: (ocean* or sea* or marine). References of all included studies are listed at the end of the flow diagram.

Studies included in qualitative synthesis
(n = 23)

Full-text articles excluded, with reasons
(n = 20)

Full-text articles assessed for eligibility
(n = 43)

Records excluded
(n = 127)

Records screened
(n = 170)

Records after duplicates removed
(n = 170)

## Identification

## Eligibility

## Included

## Screening

Studies included in quantitative synthesis (n = 23 studies, n = 96 communities, n = 1,513 unique species × treatment entries)

)

Additional records identified through other sources
(n = 4)

Records identified through database searching
(n = 166)

Beltran, Y., Cerqueda-Garcia, D., Tas, N., Thome, P. E., Iglesias-Prieto, R., & Falcon, L. I. (2016). Microbial composition of biofilms associated with lithifying rubble of Acropora palmata branches. *Fems Microbiology Ecology, 92*(1), 10. doi:10.1093/femsec/fiv162

Calbet, A., Sazhin, A. F., Nejstgaard, J. C., Berger, S. A., Tait, Z. S., Olmos, L., . . . Jakobsen, H. H. (2014). Future Climate Scenarios for a Coastal Productive Planktonic Food Web Resulting in Microplankton Phenology Changes and Decreased Trophic Transfer Efficiency. *Plos One, 9*(4), 16. doi:10.1371/journal.pone.0094388

Currie, A. R., Tait, K., Parry, H., de Francisco-Mora, B., Hicks, N., Osborn, A. M., . . . Stahl, H. (2017). Marine Microbial Gene Abundance and Community Composition in Response to Ocean Acidification and Elevated Temperature in Two Contrasting Coastal Marine Sediments. *Frontiers in Microbiology, 8*, 17. doi:10.3389/fmicb.2017.01599

Dove, S. G., Kline, D. I., Pantos, O., Angly, F. E., Tyson, G. W., & Hoegh-Guldberg, O. (2013). Future reef decalcification under a business-as-usual CO<sub>2</sub> emission scenario. *Proceedings of the National Academy of Sciences, 110*(38), 15342-15347. doi:10.1073/pnas.1302701110

Eklof, J. S., Alsterberg, C., Havenhand, J. N., Sundback, K., Wood, H. L., & Gamfeldt, L. (2012). Experimental climate change weakens the insurance effect of biodiversity. *Ecology Letters, 15*(8), 864-872. doi:10.1111/j.1461-0248.2012.01810.x

Eklof, J. S., Havenhand, J. N., Alsterberg, C., & Gamfeldt, L. (2015). Community-level effects of rapid experimental warming and consumer loss outweigh effects of rapid ocean acidification. *Oikos, 124*(8), 1040-1049. doi:10.1111/oik.01544

Feng, Y. Y., Hare, C. E., Leblanc, K., Rose, J. M., Zhang, Y. H., DiTullio, G. R., . . . Hutchins, D. A. (2009). Effects of increased pCO(2) and temperature on the North Atlantic spring bloom. I. The phytoplankton community and biogeochemical response. *Marine Ecology Progress Series, 388*, 13-25. doi:10.3354/meps08133

Goldenberg, S. U., Nagelkerken, I., Marangon, E., Bonnet, A., Ferreira, C. M., & Connell, S. D. (2018). Ecological complexity buffers the impacts of future climate on marine consumers. *Nature Climate Change, 8*(3), 229-+. doi:10.1038/s41558-018-0086-0

Hale, R., Calosi, P., McNeill, L., Mieszkowska, N., & Widdicombe, S. (2011). Predicted levels of future ocean acidification and temperature rise could alter community structure and biodiversity in marine benthic communities. *Oikos, 120*(5), 661-674. doi:10.1111/j.1600-0706.2010.19469.x

Horn, H. G., Boersma, M., Garzke, J., Loder, M. G. J., Sommer, U., & Aberle, N. (2016). Effects of high CO2 and warming on a Baltic Sea microzooplankton community. *Ices Journal of Marine Science, 73*(3), 772-782. doi:10.1093/icesjms/fsv198

Keys, M., Tilstone, G., Findlay, H. S., Widdicombe, C. E., & Lawson, T. (2018). Effects of elevated CO2 and temperature on phytoplankton community biomass, species composition and photosynthesis during an experimentally induced autumn bloom in the western English Channel. *Biogeosciences, 15*(10), 3203-3222. doi:10.5194/bg-15-3203-2018

Kim, J.-M., Lee, K., Yang, E. J., Shin, K., Noh, J. H., Park, K.-t., . . . Jang, M.-C. (2010). Enhanced Production of Oceanic Dimethylsulfide Resulting from CO2-Induced Grazing Activity in a High CO2 World. *Environmental Science & Technology, 44*(21), 8140-8143. doi:10.1021/es102028k

Meadows, A. S., Ingels, J., Widdicombe, S., Hale, R., & Rundle, S. (2015). *Effects of elevated CO2 and temperature on an intertidal meiobenthic community*. Retrieved from: https://doi.org/10.1594/PANGAEA.859078

Mensch, B., Neulinger, S. C., Graiff, A., Pansch, A., Kunzel, S., Fischer, M. A., & Schmitz, R. A. (2016). Restructuring of Epibacterial Communities on Fucus vesiculosus forma mytili in Response to Elevated pCO(2) and Increased Temperature Levels. *Frontiers in Microbiology, 7*, 15. doi:10.3389/fmicb.2016.00434

Moustaka-Gouni, M., Kormas, K. A., Scotti, M., Vardaka, E., & Sommer, U. (2016). Warming and Acidification Effects on Planktonic Heterotrophic Pico- and Nanoflagellates in a Mesocosm Experiment. *Protist, 167*(4), 389-410. doi:10.1016/j.protis.2016.06.004

Park, K. T., Lee, K., Shin, K., Yang, E. J., Hyun, B., Kim, J. M., . . . Jeong, H. J. (2014). Direct Linkage between Dimethyl Sulfide Production and Microzooplankton Grazing, Resulting from Prey Composition Change under High Partial Pressure of Carbon Dioxide Conditions. *Environmental Science & Technology, 48*(9), 4750-4756. doi:10.1021/es403351h

Sarmento, V. C., Santos, P. J. P., Hale, R., Ingels, J., & Widdicombe, S. (2017). Effects of elevated CO2 and temperature on an intertidal harpacticoid copepod community. *Ices Journal of Marine Science, 74*(4), 1159-1169. doi:10.1093/icesjms/fsw192

Sett, S., Schulz, K. G., Bach, L. T., & Riebesell, U. (2018). Shift towards larger diatoms in a natural phytoplankton assemblage under combined high-CO2 and warming conditions. *Journal of Plankton Research, 40*(4), 391-406. doi:10.1093/plankt/fby018

Sommer, U., Paul, C., & Moustaka-Gouni, M. (2014). *Mesocosm experiment on warming and acidification effects in 2012: Cell numbers*. Retrieved from: https://doi.org/10.1594/PANGAEA.840841

Troedsson, C., Bouquet, J. M., Lobon, C. M., Novac, A., Nejstgaard, J. C., Dupont, S., . . . Thompson, E. M. (2013). Effects of ocean acidification, temperature and nutrient regimes on the appendicularian Oikopleura dioica: a mesocosm study. *Marine Biology, 160*(8), 2175-2187. doi:10.1007/s00227-012-2137-9

Ullah, H., Nagelkerken, I., Goldenberg, S. U., & Fordham, D. A. (2018). Climate change could drive marine food web collapse through altered trophic flows and cyanobacterial proliferation. *Plos Biology, 16*(1), 21. doi:10.1371/journal.pbio.2003446

Walden, G., Noirot, C., & Nagelkerken, I. (2019). A future 1.2 degrees C increase in ocean temperature alters the quality of mangrove habitats for marine plants and animals. *Science of the Total Environment, 690*, 596-603. doi:10.1016/j.scitotenv.2019.07.029

Werner, F. J., Graiff, A., & Matthiessen, B. (2016). Temperature effects on seaweed-sustaining top-down control vary with season. *Oecologia, 180*(3), 889-901. doi:10.1007/s00442-015-3489-x

**a)**

**b)**

**Fig. S2** Percent species lost vs gained at (a) CO_2_ vents compared to their associated control sites in natural ecosystems, and (b) in mesocosm studies compared to their associated control mesocosms. OA = ocean acidification, T = ocean warming, OAT = ocean warming and acidification. Data points represent individual studies; dotted line represents equal percentage of lost vs gained species. Data points on the x-axis represent studies where species were only lost under climate treatments, data points on the y-axis represent studies where species were only gained, data points in the origin represent studies where no change in species numbers was observed, all other data points represent studies with species gains as well as losses. Statistical results comparing species gains vs losses are shown in Table S2.

**Fig. S3** Relative cover (square-root transformed) of taxonomic groups of sessile species at controls and their associated vent sites for the four natural ecosystems assessed. The dotted line represents the hypothetical line of no change, i.e. where the cover of a species at a vent site equals that of its associated control site; deviations from the line indicate either an increase (upward) or decrease (downward) in cover at vents. Species positioned on the x-axes and y-axes highlight species lost vs gained, respectively, under ocean acidification. CCA = crustose coralline algae, cal. = calcareous. Circles indicate individual taxa.

**Fig. S4** Comparison of density (square-root) of taxonomic groups of predominantly mobile fauna at controls and their associated vent sites for the four natural ecosystems assessed. The dotted line shows the hypothetical line of no change, i.e. where the density of a species at a vent site equals that of its associated control site; deviations from the line indicate either an increase (upward) or decrease (downward) in density at vents. Circles indicate individual taxa. Only taxa which were assessed as densities in their respective study (i.e. abundance per unit surface area) were included in this graph. Taxon abundances assessed in other units (e.g. numbers per experimental unit, relative abundance) could not be included in this graph due to the different unit scales, but they were included in all calculations of the biodiversity and community change metrics.

**Fig. S5** Relative density (%) of taxonomic groups at controls and their associated climate treatments in mesocosm studies. Relative densities were calculated as proportional density of a species to the maximum density of a species within a study. The dotted line shows the hypothetical line of no change, i.e. where the density of a species in control mesocosms equals that of its associated climate treatment; deviations from the line indicate either an increase (upward) or decrease (downward) in density in climate treatments. OA = ocean acidification, T = ocean warming, OAT = ocean warming and acidification. Circles indicate individual taxa.

Canopy reefs Non-canopy reefs Coral reefs Seagrass beds Mesocosms

**Fig. S6** Mean (+ SE) relative species richness (a–e) and community evenness (f–j) of four major benthic ecosystems at control (C) and CO_2_ vent (V) sites, and mesocosm studies under different climate treatments (C = control, OA = ocean acidification, T = ocean warming, OAT = acidification + warming). * = significant difference; ns = not significant; see statistical results in Table S4a.

**Fig. S7** Mean (± SE; left-hand panels) (a) species overlap, (b) species turnover, (c) nestedness-resultant component of β-diversity, (d) taxonomic β-diversity, (e) community dissimilarity, and (f) species dominance shifts, in natural ecosystems and in mesocosms. OA = ocean acidification, T = ocean warming, OAT = ocean warming + acidification. Data points in right-hand panels represent individual studies and their taxa studied. In the left-hand panels results of statistical tests are shown separately for the comparison among natural ecosystems, and for the comparison of mesocosm treatments. Different letters above means indicate significant differences among the natural ecosystems. – = no post hoc comparison possible due to unbalanced design of factors. ns = not significant. Detailed statistical results are shown in Table S4.

*spp. overlap*

*ss richness*

**Fig. S8** Principal coordinates analysis of the various biodiversity metrics tested for natural ecosystems and mesocosm studies, with the vectors representing the biodiversity metrics (black lines) overlayed onto the different studies (symbols). The length and direction of each vector indicates the strength and sign, respectively, of the Pearson correlation between the vector and two PCO axes. The circle is a unit circle whose positioning is not linked to that of the studies. OA = ocean acidification, T = ocean warming, OAT = ocean warming + acidification. ‘Evenness’ and ‘richness’ refer to the direction of change in evenness and richness, respectively (i.e. increase, decrease, or no change in climate treatments compared to their controls).

**Table S1**. Statistical results testing the changes in functional group composition at natural CO_2_ vents. a) Output of a randomized block design (3-way MANOVA) testing for differences in functional group composition (based on benthic cover of various sessile functional groups) between controls and their associated vent sites, followed by a SIMPER analysis showing which functional groups contributed most to these differences. b) Similar to a) but including an additional 14 studies that only studied algal functional groups (and hence due to missing data for other functional groups in these 14 studies a SIMPER analysis could not be performed). c) Outputs of 1-way ANOVAs testing for spatial differences in functional group composition, comparing the functional similarity among all control sites to that of all vent sites for each of the four studied ecosystems at global level (see Figs. 1a-d). d) Outputs of a randomized block design (2-way ANOVAs), testing for differences in primary habitat cover at vents vs controls for each ecosystem (see Figs. 1e-h). tr = treatment (control vs CO_2_ vents; fixed), ec = ecosystem (canopy, non-canopy, coral reef, seagrass; random), lo = study location (random blocking factor). Significant main effects are shown in **bold**. For a) and b) the interaction terms (tr × ec, p = 0.535 and 0.394, respectively) were pooled with the residual for a stronger main effect (post hoc pooling at p > 0.25: Winer et al. 1991). For the SIMPER analysis: Av.Abund = average abundance, Diss = dissimilarity, SD = standard deviation, Contrib% = contribution to total dissimilarity (max = 100%), Cum.% = cumulative % dissimilarity

**a) Composition of functional groups at controls vs vents (N = 26 studies)**

Source df SS MS F P

tr 1 1888.8 1888.8 4.3735 **0.0070**

ec 2 9640.4 4820.2 11.161 **0.0001**

lo 7 9747.2 1392.5 3.2242 **0.0002**

Pooled 40 17275 431.87

Total 51 48438

SIMPER analysis control (C) vs vent (V), average dissimilarity = 29.40

C V

Functional group Av.Abund Av.Abund Av.Diss c Contrib% Cum.%

erect calcareous algae 1.32 0.93 6.20 1.28 21.10 21.10

fleshy algae 1.43 1.50 4.72 1.07 16.04 37.14

CCA 1.89 1.45 4.71 1.17 16.01 53.15

calcified filter feeders 1.22 0.77 4.43 1.02 15.05 68.20

turf & biofilm 1.84 2.16 3.99 1.00 13.56 81.76

non-calcified filter feeders 1.12 0.90 3.58 1.19 12.18 93.94

vegetation 0.48 0.49 1.78 0.37 6.06 100.00

(kelp/fucoids/seagrass)

**b) Composition of functional groups at control vs vents, incl. algae-only studies (N = 40 studies)**

Source df SS MS F P

tr 1 2529.7 2529.7 5.7855 **0.0021**

ec 2 16644 8321.9 19.033 **0.0001**

lo 10 8217.6 821.76 1.8794 **0.0158**

Pooled 65 28421 437.24

Total 79 70643

**c) Similarity (spatial) of functional groups across all controls vs all vents at global level**

Canopy reefs: similarity controls vs similarity vents

Source df SS MS F P

tr 1 1865.6 1865.6 14.537 **0.0015**

Res 23 2951.7 128.34

Total 24 4817.3

Non-canopy reefs: similarity controls vs similarity vents

Source df SS MS F P

tr 1 1885.2 1885.2 6.0839 **0.0126**

Res 154 47719 309.86

Total 155 49604

Coral reefs: similarity controls vs similarity vents

Source df SS MS F P

tr 1 3182.8 3182.8 8.1878 **0.0148**

Res 18 6996.9 388.72

Total 19 10180

Seagrass: similarity controls vs similarity vents

Source df SS MS F P

tr 1 1206.0 1206.0 5.1443 **0.0430**

Res 14 3282.2 234.44

Total 15 4488.2

**d) Change in biogenic habitat cover at natural CO_2_ vents at global level**

Kelp cover (canopy reefs) control–vents

Source df SS MS F P

tr 1 4.1839 4.1839 8.1906 **0.0186**

lo 4 19.400 4.8500 9.4947 **0.0039**

Res 9 4.5973 0.5108

Total 14 27.226

Erect calcareous algae cover (non-canopy reefs) control–vents

Source df SS MS F P

tr 1 8.3683 8.3683 5.9506 **0.0261**

lo 2 5.0896 2.5448 1.8096 0.1937

Res 24 33.751 1.4063

Total 27 47.209

Coral cover (coral reefs) control–vents

Source df SS MS F P

tr 1 9.8946 9.8946 8.9648  **0.0109**

lo 4 20.582 5.1454 4.6619 **0.0163**

Res 12 13.245 1.1037

Total 17 43.721

Seagrass cover (seagrass beds) control–vents

Source df SS MS F P

tr 1 0.0042 0.0042 7.4721 **0.0494**

Res 4 0.0022 0.0006

Total 5 0.0064

**Table S2** Outputs of a randomized block design (ANOVA), testing the effect of species replacement as shown in Figs. 2 and S2 (repl: percent species gained vs species lost at natural CO_2_ vents or in mesocosms climate treatments compared to their controls; fixed), taxonomic group (ta; random factor), and location (lo; random blocking factor). Significant main effects (or their interactions) are shown in **bold**.

Canopy reefs: species gained vs lost

Source df SS MS F P

repl 1 4037.2 4037.2 29.045 **0.0298**

ta 2 110.22 55.112 0.31815 0.7320

lo 4 475.63 118.91 0.68644 0.6125

repl×ta 2 277.64 138.82 0.80138 0.4727

Res 16 2771.6 173.23

Total 25 8304.2

Non-canopy reefs: species gained vs lost

Source df SS MS F P

repl 1 3296.6 3296.6 9.4735 0.0038

ta 5 4088.4 817.68 4.7617 0.0011

lo 4 1311.0 327.75 1.9086 0.1224

repl×ta 5 2584.6 516.92 3.0102 **0.0256**

Res 78 13394 171.72

Total 93 22587

PAIR-WISE TESTS

Term 'repl×ta' for pairs of levels of factor 'repl'

Gains vs losses for individual taxa: all p ≥ 0.0854

Term 'repl×ta' for pairs of levels of factor 'ta'

Gains vs losses for individual taxa: various significant differences among species within gains and within losses (results not shown as not relevant to the treatment testing direction of diversity change)

Coral reefs: species gained vs lost

Source df SS MS F P

repl 1 47.224 47.224 0.2488 0.6428

ta 6 1642.3 273.71 2.0570 0.0919

lo 5 9092.0 1818.4 13.666 **0.0001**

repl×ta 6 1453.9 242.31 1.8211 0.1459

Res 53 7052.2 133.06

Total 71 23701

Seagrass beds: species gained vs lost

Source df SS MS F P

repl 1 266.66 266.66 1.8213 0.2521

ta 3 709.32 236.44 1.4814 0.2463

lo 1 1909.7 1909.7 11.965 **0.0019**

repl×ta 4 566.33 141.58 0.8871 0.4859

Res 25 3990.1 159.60

Total 35 7587.0

Ocean acidification in mesocosms: species gained vs lost

Source df SS MS F P

repl 1 75.224 75.224 1.6683 0.2804

ta 3 263.71 87.902 1.1845 0.3283

lo 6 374.05 62.341 0.8401 0.5467

repl×ta 4 98.044 24.511 0.3303 0.8458

Res 32 2374.8 74.211

Total 47 3390.5

Temperature in mesocosms: species gained vs lost

Source df SS MS F P

repl 1 129.57 129.57 1.3894 0.3903

ta 3 623.15 207.72 1.8010 0.1790

lo 5 170.60 34.120 0.2958 0.8961

repl×ta 4 327.16 81.789 0.7091 0.5753

Res 25 2883.4 115.34

Total 39 4236.8

Ocean acidification + temperature in mesocosms: species gained vs lost

Source df SS MS F P

repl 1 5.0916 5.0916 0.0611 0.8071

ta 4 761.88 190.47 1.4480 0.2335

lo 8 414.08 51.761 0.3935 0.9185

repl×ta 4 182.06 45.515 0.3460 0.8314

Res 34 4472.3 131.54

Total 51 5751.3

**Table S3a** Outputs of a randomized block design (ANOVA), testing the effect of climate treatments (*in situ* – tr: control vs CO_2_ vents; mesocosms – ocean acidification OA: control vs elevated CO_2_, temperature treatment T: control vs elevated; all fixed factors), taxonomic group (ta; random factor), and location (lo; random blocking factor) on relative species richness and evenness as shown in Fig. S6. Significant main effects (or their interactions) are shown in **bold**. Based on the same data, the relative change (Δ) in diversity and evenness between vents and their paired control sites is shown in Fig. 3.

**Species richness**

Canopy reefs

Source df SS MS F P

tr 1 3839.4 3839.4 27.380 **0.0326**

ta 2 26.479 13.239 0.0719 0.9308

lo 4 451.10 112.78 0.6124 0.6560

tr×ta 2 279.38 139.69 0.7585 0.4848

Res 15 2762.5 184.17

Total 24 7889.2

Non-canopy reefs

Source df SS MS F P

tr 1 3297.5 3297.5 9.4758 0.0049

ta 5 4088.4 817.68 4.7612 **0.0010**

lo 4 1310.0 327.50 1.9070 0.1221

tr×ta 5 2584.5 516.91 3.0100 **0.0218***

Res 78 13396 171.74

Total 93 22587

*a post-hoc test showed treatment-specific differences among taxa, but no treatment effects for individual taxa

Coral reefs

Source df SS MS F P

tr 1 47.224 47.224 0.2488 0.6374

ta 6 1642.3 273.71 2.0570 0.0911

lo 5 9092.0 1818.4 13.666 **0.0001**

tr×ta 6 1453.9 242.31 1.8211 0.1432

Res 53 7052.2 133.06

Total 71 23701

Seagrass beds

Source df SS MS F P

tr 1 220.95 220.95 1.2854 0.3185

ta 3 849.23 283.08 1.5395 0.2233

lo 1 1759.0 1759.0 9.5664 **0.0048**

tr×ta 4 671.05 167.76 0.9124 0.4822

Res 24 4413.0 183.87

Total 34 8194.4

Mesocosms

Source df SS MS F P

OA 1 4.9293 4.9293 0.0557 0.8320

T 1 39.576 39.576 0.3882 0.5543

ta 5 4811.3 962.26 6.0969 **0.0003**

lo 8 1784.1 223.01 1.4130 0.2075

OA×T 1 241.24 241.24 1.8184 0.2653

OA×ta 5 115.56 23.113 0.1465 0.9775

T×ta 5 246.51 49.302 0.3124 0.8902

OA×T×ta 5 551.32 110.26 0.6986 0.6121

Res 64 10101 157.83

Total 95 17562

**Evenness**

Canopy reefs

Source df SS MS F P

tr 1 0.0196 0.0196 47.872  **0.0170**

ta 2 0.1219 0.0609 83.992 **0.0001**

lo 4 0.3159 0.0790 108.88 **0.0001**

tr×ta 2 0.0008 0.0004 0.5595 0.5799

Res 15 0.0109 0.0007

Total 24 0.3582

Non-canopy reefs

Source df SS MS F P

tr 1 0.1393 0.1393 6.3776 **0.0403**

ta 5 1.6489 0.3298 9.5683 **0.0001**

lo 4 0.2015 0.0504 1.4612 0.2223

tr×ta 5 0.0487 0.0097 0.2826 0.9224

Res 78 2.6883 0.0344

Total 93 4.8819

Coral reefs

Source df SS MS F P

tr 1 0.0007 0.0007 0.0198 0.8971

ta 6 0.3176 0.0529 1.3556 0.2505

lo 5 0.1935 0.0387 0.9909 0.4313

tr×ta 6 0.1946 0.0324 0.8305 0.5551

Res 53 2.0699 0.0391

Total 71 3.0826

Seagrass beds

Source df SS MS F P

tr 1 0.0025 0.0025 0.1489 0.7236

ta 3 0.5151 0.1717 6.0058 **0.0034**

lo 1 0.0359 0.0359 1.2570 0.2675

tr×ta 4 0.0512 0.0128 0.4474 0.7697

Res 24 0.6861 0.0286

Total 34 1.2563

Mesocosms

Source df SS MS F P

OA 1 0.0008 0.0008 0.0461 0.8336

T 1 0.0159 0.0159 0.7632 0.4498

ta 5 1.1233 0.2247 7.9368 **0.0001**

lo 8 0.7576 0.0947 3.3453 **0.0035**

OA×T 1 0.0074 0.0074 0.4080 0.5855

OA×ta 5 0.0275 0.0055 0.1942 0.9646

T×ta 5 0.0686 0.0137 0.4848 0.7754

OA×T×ta 5 0.0446 0.0089 0.3151 0.8939

Res 64 1.8116 0.0283

Total 95 3.8490

**Table S3b** Outputs of a randomized block design (ANOVA), testing the effect of direction of change (ch: increase vs no change vs decrease; fixed), taxonomic group (ta; random factor), and location (lo; random blocking factor) on species richness and evenness of species communities as shown in Fig. 3. Significant main effects (or their interactions) are shown in **bold**. For the post-hoc tests only significant results are displayed (all others p > 0.05).

**Species richness**

Canopy reefs

Source df SS MS F P

ch 2 6.9714 3.4857 56.643 **0.0012**

ta 2 < 0.0001 < 0.0001 < 0.0001 1.0000

lo 4 < 0.0001 < 0.0001 Negative

ch×ta 4 0.2462 0.0615 1 0.5052

Res 26 1.6000 0.0615

Total 38 8.6667

PAIR-WISE TESTS

Groups t P

**decrease > increase 6.2666 0.0251**

decrease > no change 13.497 **0.0061**

Non-canopy reefs

Source df SS MS F P

ch 2 2.4139 1.2070 3.4338 0.0793

ta 5 < 0.0001 < 0.0001 Negative

lo 4 < 0.0001 < 0.0001 < 0.0001 1.0000

ch×ta 10 5.1681 0.5168 2.8873 **0.0027**

Res 119 21.300 0.1790

Total 140 31.333

PAIR-WISE TESTS

Term 'tr×ta' for pairs of levels of factor 'treatment'

Within level 'algae' of factor 'taxon'

Groups t P

decrease > increase 3.6776 **0.0065**

Within level 'inverts' of factor 'taxon'

Groups t P

**decrease > increase 2.4121 0.0269**

no change > increase 3.6181 **0.0054**

Coral reefs

Source df SS MS F P

ch 2 1.1788 0.5894 2.2233 0.1621

ta 6 < 0.0001 < 0.0001 Negative

lo 5 < 0.0001 < 0.0001 Negative

ch×ta 12 3.8746 0.3229 1.5925 0.1041

Res 82 16.625 0.2028

Total 107 24.000

Seagrass beds

Source df SS MS F P

ch 2 1.702 0.8510 2.9430 0.1194

ta 3 < 0.0001 < 0.0001 < 0.0001 1.0000

lo 1 < 0.0001 < 0.0001 < 0.0001 1.0000

ch×ta 8 2.5333 0.3167 1.4795 0.1916

Res 38 8.1333 0.2140

Total 53 12.000

Ocean acidification in mesocosms

Source df SS MS F P

ch 2 2.2240 1.1120 4.2206 0.0689

ta 4 < 0.0001 < 0.0001 < 0.0001 1.0000

lo 6 < 0.0001 < 0.0001 < 0.0001 1.0000

ch×ta 10 3.3667 0.3367 1.7856 0.0713

Res 48 9.0500 0.1885

Total 71 16.000

Temperature in mesocosms

Source df SS MS F P

ch 2 1.7079 0.8539 2.6692 0.1460

ta 4 < 0.0001 < 0.0001 Negative

lo 5 < 0.0001 < 0.0001 Negative

ch×ta 10 4.1833 0.4183 2.2378 **0.0280**

Res 37 6.9167 0.1869

Total 59 13.333

PAIR-WISE TESTS

Within level 'inverts' of factor 'taxon'

Groups t P

no change > decrease 2.8322 **0.0345**

no change > increase 2.8322 **0.0297**

Ocean acidification + temperature in mesocosms

Source df SS MS F P

ch 2 1.0380 0.5190 2.0968 0.2025

ta 5 < 0.0001 < 0.0001 < 0.0001 1.0000

lo 8 < 0.0001 < 0.0001 < 0.0001 1.0000

ch×ta 10 2.2308 0.2231 0.8286 0.6407

Res 52 14.000 0.2692

Total 77 17.333

**Evenness**

Canopy reefs

Source df SS MS F P

ch 2 8.5714 4.2857 Denominator is 0

ta 2 < 0.0001 < 0.0001 Denominator is 0

lo 4 < 0.0001 < 0.0001 Denominator is 0

ch×ta 4 < 0.0001 < 0.0001 Denominator is 0

Res 26 < 0.0001 < 0.0001

Total 38 8.6667

No test possible because all studies showed a decrease (i.e. only 0-values for increase and no change, and therefore no within-group variance)

Non-canopy reefs

Source df SS MS F P

ch 2 7.4153 3.7077 19.240 0.0005

ta 5 < 0.0001 < 0.0001 Negative

lo 4 < 0.0001 < 0.0001 < 0.0001 1.0000

ch×ta 10 2.5390 0.2539 1.9705 **0.0428**

Res 119 15.333 0.1289

Total 140 31.333

PAIR-WISE TESTS

Term ' ch×ta' for pairs of levels of factor 'treatment'

Within level 'algae' of factor 'taxon'

Groups t P

decrease > no change 6.6108 **0.0011**

**decrease > increase 3.7607 0.0032**

Within level 'inverts' of factor 'taxon'

Groups t P

decrease > no change 2.9814 **0.0121**

increase > no change 2.9814 **0.0137**

Within level 'microbes' of factor 'taxon'

Groups t P

decrease > no change 3.9340 **0.0061**

increase > no change 2.3604 **0.0305**

Within level 'miscellaneous' of factor 'taxon'

Groups t P

decrease > no change 10.393 **0.0001**

**decrease > increase 6.5713 0.0001**

Within level 'polychaetes' of factor 'taxon'

Groups t P

decrease > no change 4.5826 **0.0006**

**decrease > increase 2.1602 0.0455**

Coral reefs

Source df SS MS F P

ch 2 2.9552 1.4776 4.8925 0.0353

ta 6 < 0.0001 < 0.0001 Negative

lo 5 < 0.0001 < 0.0001 Negative

ch×ta 12 5.4714 0.4560 3.3563 **0.0003**

Res 82 11.140 0.1359

Total 107 24.000

PAIR-WISE TESTS

Term 'ch×ta' for pairs of levels of factor 'treatment'

Within level 'algae' of factor 'taxon'

Groups t P

increase > no change 2.7768 **0.0307**

Within level 'corals' of factor 'taxon'

Groups t P

decrease > no change 4.4096 **0.0129**

Within level 'inverts' of factor 'taxon'

Groups t P

decrease > increase Denominator is 0

No test possible because all studies show a decrease (i.e. only 0-values for increase and no change, and therefore no within-group variance)

Within level 'microbes' of factor 'taxon'

Groups t P

decrease, > no change 2.5298 **0.0231**

increase > no change 3.1623 **0.0054**

Within level 'miscellaneous' of factor 'taxon'

Groups t P

decrease > no change 3.8431 **0.0261**

Seagrass beds

Source df SS MS F P

ch 2 2.9793 1.4896 6.0109 **0.0365**

ta 3 < 0.0001 < 0.0001 < 0.0001 1.0000

lo 1 < 0.0001 < 0.0001 Negative

ch×ta 8 2.2667 0.2833 1.8779 0.0825

Res 38 5.7333 0.1509

Total 53 12.000

PAIR-WISE TESTS

Term 'tr'

Groups t P

increase > no change 4.732 **0.0165**

Ocean acidification in mesocosms

Source df SS MS F P

ch 2 1.5390 0.7695 2.8646 0.1272

ta 4 < 0.0001 < 0.0001 < 0.0001 1.0000

lo 6 < 0.0001 < 0.0001 < 0.0001 1.0000

ch×ta 10 3.3667 0.3367 1.6921 0.0856

Res 48 9.5500 0.1990

Total 71 16.000

Temperature in mesocosms

Source df SS MS F P

ch 2 1.6561 0.8281 4.2377 0.0617

ta 4 < 0.0001 < 0.0001 Negative

lo 5 < 0.0001 < 0.0001 Negative

ch×ta 10 1.8500 0.1850 0.8832 0.5697

Res 37 7.7500 0.2095

Total 59 13.333

Ocean acidification + temperature in mesocosms

Source df SS MS F P

ch 2 1.7697 0.8849 4.5735 0.0551

ta 5 < 0.0001 < 0.0001 Negative

lo 8 < 0.0001 < 0.0001 Negative

ch×ta 10 1.7423 0.1742 0.8274 0.6348

Res 52 10.950 0.2106

Total 77 17.333

**Table S4** Outputs of a randomized block design (ANOVA), testing the effect of system (sy: 4 habitats for vents and 3 climate treatments for mesocosms, respectively; fixed factor), taxonomic group (ta; random factor nested within system), and location (lo; random blocking factor) on the various biodiversity and community-change metrics at in situ CO_2_ vents and mesocosms as shown in Fig. S7. Significant effects are shown in **bold**.

Species overlap in situ (log-transformed)

Source df SS MS F P

sy 3 8.8876 2.9625 10.836 **0.0081**

lo 12 41.693 3.4744 51.644 **0.0001**

ta(sy) 17 12.551 0.7383 10.974 **0.0001**

Res 81 5.4494 0.0673

Total 113 68.627

Species overlap mesocosms (log-transformed)

Source df SS MS F P

sy 2 0.0005 0.0002 0.0258 0.9644

lo 8 0.1078 0.0135 2.1058 0.0742

ta(sy) 15 0.1682 0.0112 1.7521 0.0898

Res 44 0.2815 0.0064

Total 69 0.5098

Species turnover in situ

Source df SS MS F P

sy 3 0.5516 0.1839 4.5587 **0.0410**

lo 12 2.8396 0.2366 13.370 **0.0001**

ta(sy) 17 1.5534 0.0914 5.1626 **0.0001**

Res 81 1.4336 0.0177

Total 113 6.6366

Species turnover mesocosms

Source df SS MS F P

sy 2 0.0006 0.0003 0.2080 0.7698

lo 5 0.0250 0.0050 1.5289 0.2078

ta(sy) 2 0.0015 0.0007 0.2257 0.7540

Res 56 0.1835 0.0033

Total 69 0.2181

Species nestedness in situ (fourth root-transformed)

Source df SS MS F P

sy 3 0.9833 0.32775 3.8222 **0.0496**

lo 12 1.8366 0.15305 2.2146 **0.0126**

ta(sy) 17 2.0958 0.12328 1.7839 **0.0436**

Res 81 5.5979 0.06911

Total 113 11.821

Species nestedness mesocosms (fourth root -transformed)

Source df SS MS F P

sy 2 0.0011 0.0006 0.0061 0.9931

lo 8 1.0139 0.1267 2.6183 **0.0164**

ta(sy) 15 1.7122 0.1142 2.3582 **0.0081**

Res 44 2.1299 0.0484

Total 69 4.4438

β-diversity in situ

Source df SS MS F P

sy 3 0.1162 0.0387 0.7956 0.4783

lo 12 2.5577 0.2131 7.1524 **0.0001**

ta(sy) 17 1.5512 0.0912 3.0620 **0.0006**

Res 81 2.4138 0.0298

Total 113 7.9384

β-diversity mesocosms

Source df SS MS F P

sy 2 0.0003 0.0002 0.0220 0.9706

lo 8 0.0886 0.0111 2.1460 0.0626

ta(sy) 15 0.1403 0.0094 1.8109 0.0678

Res 44 0.2272 0.0052

Total 69 0.4161

Community dissimilarity in situ

Source df SS MS F P

sy 3 603.08 201.03 0.8043 0.5055

lo 12 7747.3 645.61 4.3482 **0.0002**

ta(sy) 17 8139.4 478.79 3.2247 **0.0094**

Res 81 12027 148.48

Total 113 35156

Community dissimilarity mesocosms

Source df SS MS F P

sy 2 224.74 112.37 0.3495 0.7306

lo 8 1186.6 148.33 0.9359 0.4893

ta(sy) 15 6271.2 418.08 2.6380 **0.0079**

Res 44 6973.2 158.48

Total 69 15041

Dominance shift in situ

Source df SS MS F P

sy 3 0.0833 0.0278 2.1588 0.1829

lo 12 0.2419 0.0202 1.8348 0.0671

ta(sy) 17 0.2910 0.0171 1.5585 0.1000

Res 81 0.8897 0.0110

Total 113 1.5506

Dominance shift mesocosms

Source df SS MS F P

sy 2 0.0069 0.0034 0.4259 0.6713

lo 8 0.1179 0.0147 1.6977 0.1296

ta(sy) 15 0.1152 0.0077 0.8848 0.5808

Res 44 0.3819 0.0087

Total 69 0.6742

**Table S5**. Statistical results showing outputs of simple linear regression between: a) various biodiversity and community-change metrics and change in habitat cover for each of the four ecosystems, and between change in major habitat cover and degree of experimental pH change (Δ pH) between controls and their respective CO_2_ vent sites, and linear regressions between b) various biodiversity and community-change metrics and magnitude of experimental pH, *p*CO_2_ and temperature change (Δ). Note that for the regressions below a reduced data set was used: a) only including studies for which change in habitat cover was available, and b) only including studies for which levels of experimental climate stressors were available. For all statistical tests other than in this Table, the full data set of biodiversity metrics was used. NA_1_ = not applicable (species turnover = 0), NA_2_ = not applicable (no variance across samples), – = negative relationship. Significant p-values are shown in **bold**.

|  | 1. **Habitat change (% cover)** | | | | | | | |  | 1. **Magnitude of climate stress** | | | | | |
| --- | --- | --- | --- | --- | --- | --- | --- | --- | --- | --- | --- | --- | --- | --- | --- |
|  | Canopy reefs | | Non-canopy reefs | | Coral reefs | | Seagrass beds | |  | Δ pH | | Δ *p*CO_2_ | | Δ Temperature | |
|  | R^2^ | p-value | R^2^ | p-value | R^2^ | p-value | R^2^ | p-value |  | R^2^ | p-value | R^2^ | p-value | R^2^ | p-value |
| Community dissimilarity | **0.621** | **0.020** | 0.211 | 0.099 | 0.084 | 0.448 | –0.992 | 0.056 |  | 0.094 | **< 0.001** | < 0.001 | 0.940 | 0.043 | **0.005** |
| Δ Evenness | < 0.001 | 0.975 | 0.013 | 0.694 | –0.166 | 0.277 | 0.065 | 0.836 |  | –0.012 | 0.159 | –0.012 | 0.371 | –0.043 | **0.005** |
| Δ Species richness | 0.064 | 0.544 | –0.032 | 0.538 | < 0.001 | 0.975 | –0.973 | 0.106 |  | 0.080 | **< 0.001** | –0.001 | 0.846 | –0.008 | 0.227 |
| Species overlap | –0.172 | 0.307 | –0.003 | 0.849 | 0.087 | 0.441 | –0.973 | 0.106 |  | 0.138 | **< 0.001** | 0.001 | 0.773 | 0.079 | **< 0.001** |
| β-diversity | 0.172 | 0.307 | 0.003 | 0.849 | –0.087 | 0.441 | 0.973 | 0.106 |  | 0.138 | **< 0.001** | 0.001 | 0.776 | 0.079 | **< 0.001** |
| Species nestedness | –0.099 | 0.448 | 0.153 | 0.166 | –0.210 | 0.215 | 0.973 | 0.106 |  | 0.059 | **0.002** | 0.006 | 0.513 | 0.043 | **0.005** |
| Species turnover | 0.227 | 0.232 | –0.035 | 0.520 | –0.040 | 0.605 | NA_1_ | NA_1_ |  | 0.077 | **< 0.001** | 0.016 | 0.297 | 0.037 | **0.008** |
| Dominance shift | –0.158 | 0.329 | –0.241 | 0.075 | –0.086 | 0.445 | 0.972 | 0.108 |  | 0.013 | 0.137 | 0.001 | 0.760 | 0.036 | **0.010** |
| Evenness change | NA_2_ | NA_2_ | –0.000 | 0.948 | 0.007 | 0.827 | 0.973 | 0.106 |  | –0.018 | 0.084 | < 0.001 | 0.911 | –0.060 | **< 0.001** |
| Richness change | NA_2_ | NA_2_ | –0.144 | 0.181 | –0.026 | 0.676 | 0.122 | 0.773 |  | 0.021 | 0.062 | –0.004 | 0.593 | –0.010 | 0.176 |
| Δ pH | –0.168 | 0.361 | 0.028 | 0.569 | –0.005 | 0.863 | < -0.001 | 0.991 |  |  |  |  |  |  |  |
